# Overview on the complexity of androgen receptor-targeted therapy for prostate cancer

**DOI:** 10.1186/s12935-014-0153-1

**Published:** 2015-02-04

**Authors:** Ammad Ahmad Farooqi, Fazlul H Sarkar

**Affiliations:** Laboratory for Translational Oncology and Personalized Medicine, Rashid Latif Medical College, 35 Km Ferozepur Road, Lahore, Pakistan; Departments of Pathology and Oncology, Karmanos Cancer Institute, Wayne State University School of Medicine, 740 HWCRC, 4100 JohnR Street, Detroit, MI 48201 USA

**Keywords:** Prostate cancer, Intracellular signaling, Apoptosis

## Abstract

In the past decades, the field of prostate cancer (PCa) biology has developed exponentially and paralleled with that has been the growing interest in translation of laboratory findings into clinical practice. Based on overwhelming evidence of high impact research findings which support the underlying cause of insufficient drug efficacy in patients progressing on standard androgen deprivation therapy (ADT) is due to persistent activation of the androgen receptor (AR) signaling axis. Therefore, newer agents must be discovered especially because newer ADT such as abiraterone and enzalutamide are becoming ineffective due to rapid development of resistance to these agents. High-throughput technologies are generating massive and highly dimensional genetic variation data that has helped in developing a better understanding of the dynamic repertoire of AR and AR variants. Full length AR protein and its variants modulate a sophisticated regulatory system to orchestrate cellular responses. We partition this multicomponent review into subsections addressing the underlying mechanisms of resistance to recent therapeutics, positive and negative regulators of AR signaling cascade, and how SUMOylation modulates AR induced transcriptional activity. Experimentally verified findings obtained from cell culture and preclinical studies focusing on the potential of natural agents in inhibiting mRNA/protein levels of AR, nuclear accumulation and enhanced nuclear export of AR are also discussed. We also provide spotlight on molecular basis of enzalutamide resistance with an overview of the strategies opted to overcome such resistance. AR variants are comprehensively described and different mechanisms that regulate AR variant expression are also discussed. Reconceptualization of phenotype- and genotype-driven studies have convincingly revealed that drug induced resistance is a major stumbling block in standardization of therapy. Therefore, we summarize succinctly the knowledge of drug resistance especially to ADT and potential avenues to overcome such resistance for improving the treatment outcome of PCa patients.

## Introduction

Androgen receptor (AR) blockers have been incorporated in the backbone of PCa therapeutics. Mutation and amplification of AR gene, growth factor mediated signaling induced activation of AR, overexpression of nuclear receptor co-activators, alternative splicing variants and steroid metabolism enzymes are widely studied biological mechanisms that underpin castration resistant prostate cancer (CRPC) phenotype. Substantial fraction of information has been added into the existing pool of knowledge, and dysregulation of intracellular signaling networks in PCa lends credence to the mantra that increasing molecular understanding is catalyzing the generation of AR antagonists. Mounting evidence has provided a platform to investigate systematically not only the molecular complexity of CRPC, leading to the identification of disease pathways and modules, but also changes in the recruitment and regulatory effect of AR on transcription as PCa progresses.

First generation AR antagonists such as bicalutamide and nilutamide gained appreciation but agonistic properties were noted in AR over-expressing PCa cells, which raised some concerns for the use of these agents in human PCa patients. Whereas, enzalutamide was reported to be effective against AR over-expressing PCa cells and proved to be a valuable addition in the arsenal of molecular therapeutics for treating PCa after approval in 2012 by US FDA. VCaP prostate cancer cells have endogenously amplified AR gene and enzalutamide exerted inhibitory effects on proliferation of VCaP cells. Moreover, the activity of mutation bearing AR (W741C) was also notably reduced upon treatment with enzalutamide. Bicalutamide induced AR conformational change facilitated its interaction with FxxLF or LxxLL motif-containing co-activators. On the contrary, enzalutamide did not promote AR interaction with co-activators. We will now address some specific example of AR activation in the context of CRPC and subsequent metastasis (mCRPC) of PCa.

## Current treatment options for PCa patients

Lupron and other LHRH agonists are worthwhile options as part of ADT for PCa patients. Using multipronged approach, by combining antagonists such as bicalutamide with Lupron can prevent its harmful side effects [1]. There are now more treatment modalities as summarized below such as ARN-509, enzalutamide and abiraterone and their pros and cons are being discussed.

Lower treatment dose of ARN-509 and absence of any risk of seizures in patients as compared to enzalutamide treatment makes it a potent anti-androgen than enzalutamide. However, F876L mutant AR carrying cancer cell lines did not show any response to ARN-509. Moreover, F876L mutant AR was identified in plasma DNA from ARN-509-treated patients with progressive CRPC [2].

CYP17 is also chemically inhibited by Orteronel, also known as TAK-700. Considerably higher specificity for 17,20-lyase than for 17α-hydroxylase by Orteronel is a hallmark feature that differentiates it from abiraterone. More importantly, Orteronel associated manageable toxicities and its chronic administration without steroids makes it a drug of choice. Orteronel induced durable declines in PSA in mCRPC patients [3].

Although there are encouraging advancements in our understanding related to the intricacies that mediate androgen metabolism and thereby regulate AR activation [4], we still need in-depth analysis and identification of agents perhaps natural agents (no known toxicity in human) that can overcome resistance against current and next-generation AR targeting agents.

## Positive and negative regulators of AR and AR induced signaling

**Figure 1:**
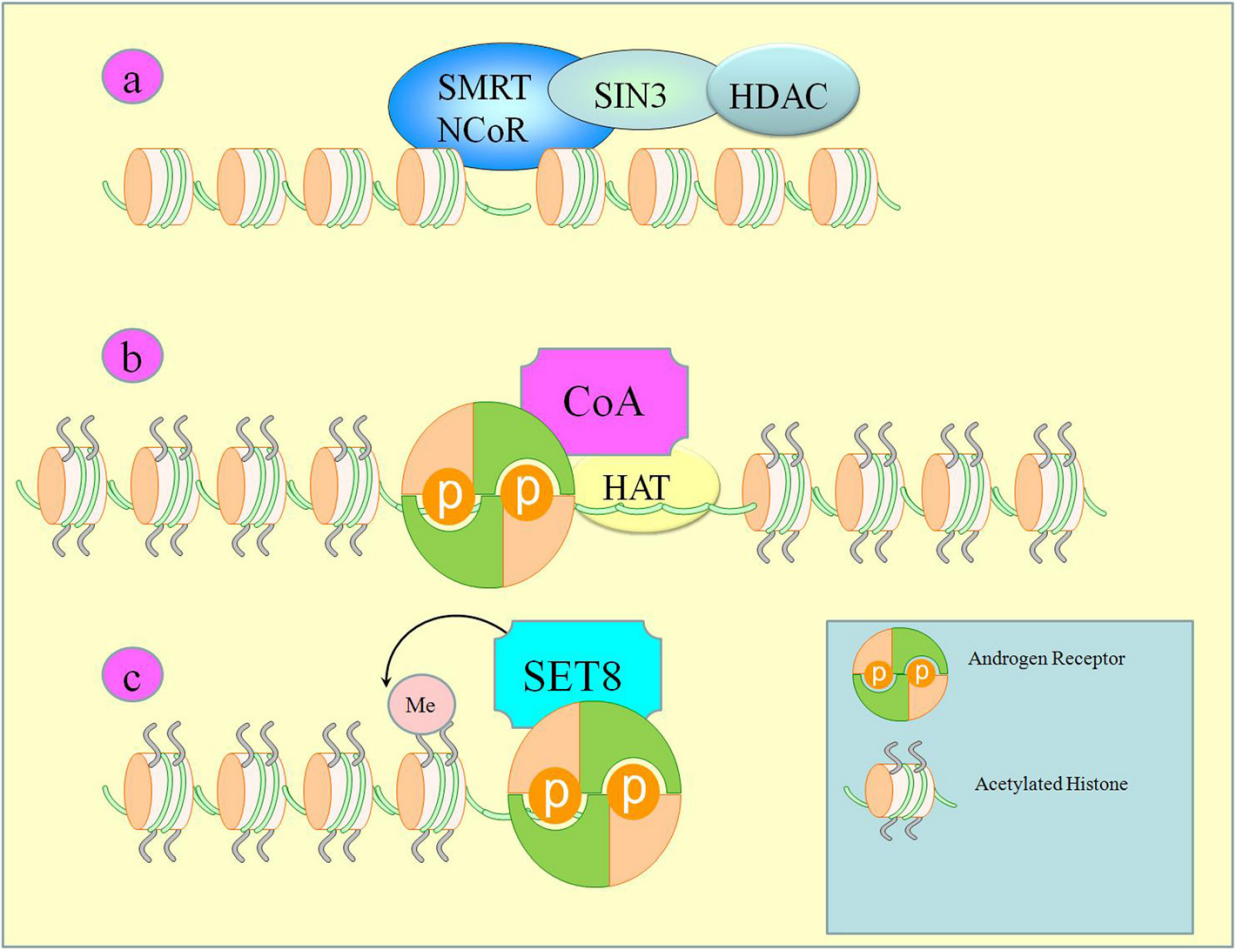
**Showing inactive and active states. **a) Co-repressors and HDAC are recruited in the absence of AR. b) AR induced positioning of co-activators, Histone Acetyltransferases. c) SET8 a histone Methytransferase co-exists with AR.

Substantial information has been added into the landscape of co-activator/co-repressor complexes associated with AR (Figure 1), and it is now known that AR interacted with phosphorylated heterogeneous nuclear ribonucleoprotein K (hnRNP K) in nuclear matrix region in PCa cells. However, astonishingly, bicalutamide treatment reduced phosphorylated hnRNP K levels and induced its dissociation from AR [5]. Nuclear accumulation of AR is notably reduced by HepaCAM in PCa cells [6]. SPOP E3 ubiquitin ligase has been shown to degrade full-length AR by recognizing a Ser/Thr-rich degron in its hinge domain [7].

MID1 is a microtubule-associated ubiquitin E3 ligase frequently overexpressed in PCa cells. MID1 bound purine-rich repeats regions in AR mRNA have been identified experimentally. Targeted inhibition of MID1 in AR overexpressing DuCaP cells notably reduced decreased protein levels of AR [8]. Skp2 (S-phase kinase-associated protein 2) is an E3 ubiquitin ligase reported to enhance AR degradation via ubiquitination. K847R mutant AR did not show Skp2 mediated ubiquitination at the mutant residue [9,10]. SET8 is a methyltransferase reported to methylate Histone H4 Lys 20 of the promoter of AR target gene PSA. Androgen stimulation promoted enrichment of SET8 at promoter region of AR target gene where it co-existed with AR [11] (Figure 1).

Jumonji domain-containing demethylase, KDM4C is a well known protein reported to enhance AR mediated transcriptional activity by modulating H3K9 demethylation. Interfering with KDM4C in LNCaP cells dramatically impaired AR induced transcriptional up-regulation of TMPRSS2, KLK3 and KLK2. SD70 contains an 8-hydroxyquinoline moiety that is an Fe(II) chelator with considerable activity against KDM4. SD70 treated PCa cells and displayed a marked decrease in AR target gene expression [12,13].

## AR induced signaling as targets for PCa therapy

### Small Ubiquitin-Related Modifier (SUMO) mediated control of AR and associated protein network as target for therapy

This section reviews mounting research work summarizing information on alteration in transcriptional network of sumoylated AR, various ways in which AR and its associated proteins are SUMOylated and how SENP-1 deconjugates SUMO from AR to enhance AR induced expression of target genes. Targeting of SENP-1 using chemical and synthetic agents is also discussed.

**Figure 2:**
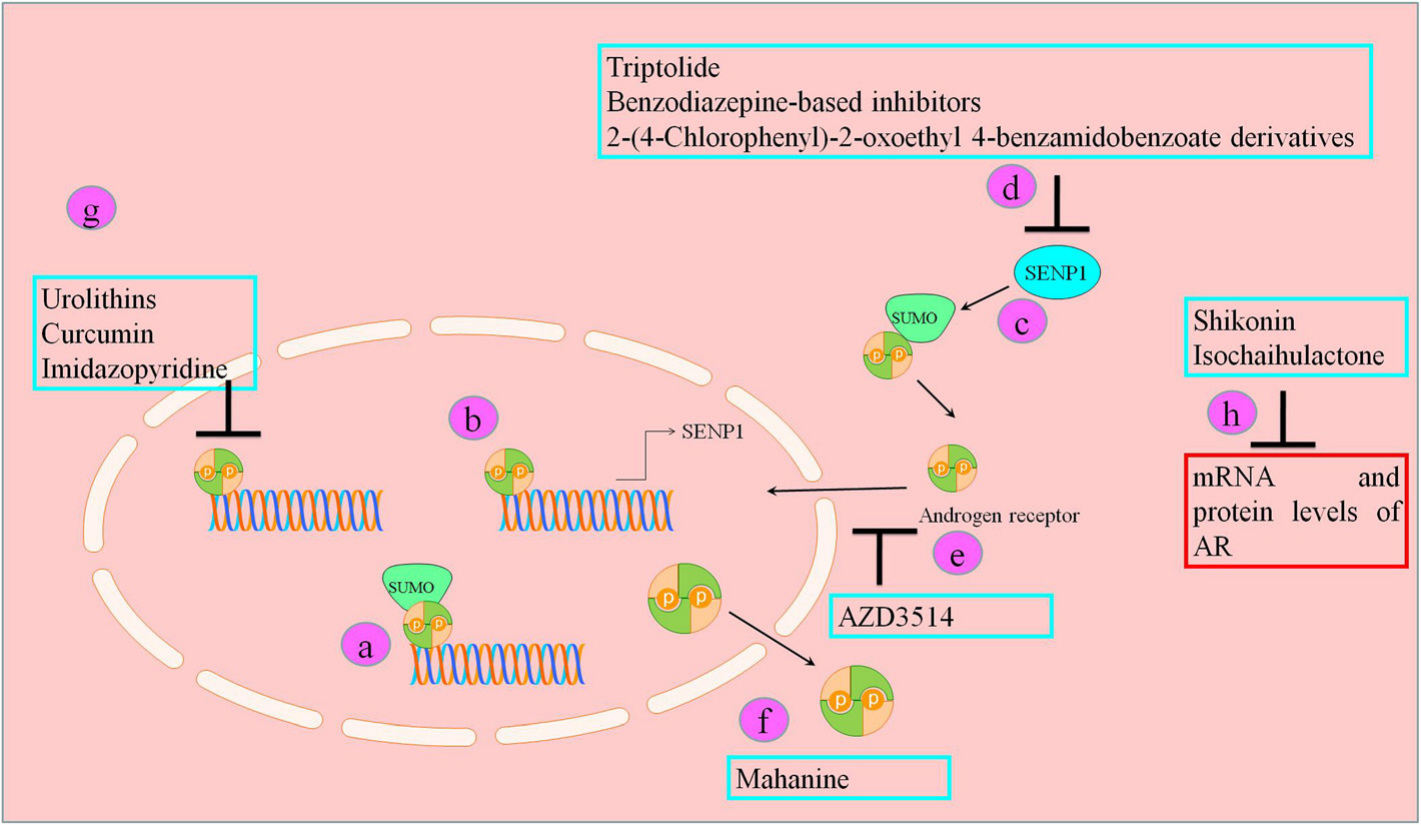
**Natural and synthetic agents mediated targeting of AR. **a) SUMOylated AR mediated transcriptional activity activity is reduced. b-c) SENP-1 is a target of AR that deconjugated SUMO from AR thus enhancing AR mediated transcriptional activity. d) Natural and synthetic agents have shown potent inhibitory effects on SENP-1. e) AZD3514 inhibited nuclear accumulation of AR. f) Mahanine promoted nuclear export of AR. g) Urolithins and Curcumin have shown to reduce AR induced transcriptional activity. h) Natural agents also decrease mRNA and protein levels of AR.

It has previously been convincingly revealed that N-terminal transactivation domain of AR undergoes covalent modification by SUMOs at two conserved lysine that may influence AR efficiency in stimulating expression of target genes. Increasingly it is being realized that SUMOylated AR shows a lower transcriptional activity (Figure 2). It is noteworthy that PC-3 cells stably expressing doubly SUMOylation site-mutated AR displayed a higher apoptotic rate [14,15]. 15-deoxy-Δ(12,14)-prostaglandin J(2) is also reported to trigger SUMOylation of AR [16]. In accordance with significant roleplay of SUMOylation, it has also been reported that mutation of the FOXA1 SUMOylation sites considerably reduced AR nuclear mobility. Forkhead box (FOX) protein A1 is involved in chromatin remodelling and facilitates AR positioning at DNA [14,15]. Gly residue is present at 524^th^ position downstream to the core of the second synergy control motif in AR. AR bearing the G524D substitution displayed a higher transcriptional activity than wild type AR. Moreover, SUMOylation-deficient AR mutant (K386R/K520R), also induced significant target gene expression [17]. Heat stress resulted in generation of prominent intranuclear granules that contained SUMO-2/3 and AR. Stress induced SUMOylated AR transportation towards nuclear matrix. Quantitative chromatin immunoprecipitation (qChIP) assay has provided clear evidence of detachment of androgen-loaded AR from regulatory regions of these genes in Heat stressed prostate cancer cells. Using anti-SUMO-2/3 antibody, it was shown that heat stressed prostate cancer cells showed accumulation of SUMO-2/3 in the AR-binding regions of the target genes [18]. Rapidly emerging experimental findings are also providing new information regarding positive regulators of AR induced signaling. SUMO-specific protease 1 (SENP1) has previously been identified as an AR regulated gene and reportedly decreased SUMOylation of AR via deconjugation of AR-SUMO covalent bond [19] (Figure 2). Triptolide, isolated from Tripterygium wilfordii Hook notably inhibited SENP1 that consequently resulted in a markedly reduced SUMOylated AR induced transcriptional activity [20]. Benzodiazepine-based inhibitors and 2-(4-Chlorophenyl)-2-oxoethyl 4-benzamidobenzoate derivatives have shown potential in targeting of SENP1 [21,22] (Figure 2). These recent reports suggest that newer arsenals are beginning to emerge for AR-targeted therapeutics which could be useful in improving the therapeutic outcome of PCa patients especially for patients with CRPC and/or mCRPC.

## Natural and Synthetic agents mediated targeting of AR for PCa therapy

Here we will provide some examples of a number of natural agents or their synthetic analog as AR-targeted therapeutics. Epibrassinolide (EBR) is a member of the brassinosteroids (BR) with notable activity against LNCaP prostate cancer cells expressing functional AR. EBR exerted its biological effects via activation of polyamine catabolic machinery in prostate cancer cells. Interfering with spermidine-spermineacetyltransferase (SSAT), an essential enzyme in polyamine catabolic machinery, dramatically impaired EBR induced apoptosis [23].

Urolithins, Walnut polyphenol metabolites have been shown to inhibit AR mediated upregulation of PSA in LNCaP cells (Figure 2). Data obtained from EMSA revealed that AR did not bind to response elements of PSA after treatment with Urolithins. Moreover, levels of Bcl-2, an anti-apoptotic protein were reduced significantly [24]. Shown in Table 1.

**Table 1:** **List of natural agents with notable activity against AR in prostate cancer**

**Agent**	**Targets**	**Reference**
Urolithins (Walnut polyphenol metabolites)	PSA ↓	Sánchez-González et al. [24]
	Bcl-2 ↓	
	AR inhibition	
Curcumin	AR inhibition	Zhou et al. [25]
Imidazopyridine	PI3K/Akt ↓	Muniyan et al. [26]
	AR induced signaling ↓	
Atraric acid	Inhibition of intramolecular amino/carboxy (N/C)-terminal interaction of the AR	Hessenkemper et al. [27]
Furanoditerpenoid spongia-13(16),-14-dien-19- oic acid		Yang et al. [28]
AZD3514	AR ↓	Loddick et al. [31]
*Rosmarinus officinalis* extract	AR ↓	Petiwala et al. [29]
Betulinic acid	AR ↓	Reiner et al. [30]
Isochaihulactone	AR mRNA ↓	Liu et al. [33,34]
Shikonin	AR protein ↓	Jang et al. [32]

Arrow indicates inhibition and/or downregulation of target proteins.

Curcumin analogues efficiently inhibited AR activity in prostate cancer cells [25].

Imidazopyridine derivatives have been shown to inhibit both PI3K/Akt and AR induced signaling in LNCaP cells [26]. Atraric acid (AA) isolated from bark of *Pygeum africanum* considerably blocked intramolecular amino/carboxy (N/C)-terminal interaction of the AR [27]. Furanoditerpenoid spongia-13(16),-14-dien-19- oic acid has been shown to block essential N/C interactions required for AR transcriptional activity. It was also noted to effectively bind ligand binding domain of AR [28]. Shown in Table 1.

C/EBP homologous protein was noted to be an essential regulator of AR degradation in *Rosmarinus officinalis* extract-treated 22Rv1 and LNCaP cells [29]. Levels of AR were reduced in LNCaP and in AR transfected PC3/DU145 cells upon treatment with plant-derived small molecule Betulinic acid (BA) [30]. Shown in Table 1.

AZD3514, an orally bioavailable drug has potent activity against AR induced signaling. AZD3514 exerted inhibitory effects on AR expression and ligand-driven nuclear accumulation of AR [31]. Shown in Table 1. Shikonin, a natural naphthoquinone isolated from Zi Cao (gromwell) repressed mRNA and protein expression of AR [32] (Figure 2). Shown in Table 1. Racemic forms of natural compound isochaihulactone also inhibited AR mRNA and protein expression [33,34] (Figure 2). It has recently been persuasively revealed that genistein, a soy phytoestrogen induced apoptosis in T877A mutant AR expressing LNCaP cells at higher doses. Moreover T877A, W741C and H874Y expressing PC-3 cells also displayed similar responses upon treatment with higher doses of genistein [35]. More importantly, it is noteworthy that a natural agent isolated from leaves of *Murraya koenigii* promoted shuttling of AR from nucleus to cytoplasm. Ligand-induced AR phosphorylation at Ser-81 was also notably repressed in Mahanine treated prostate cancer cells [36]. Novel Nor-Homo- and Spiro-Oxetan- Steroids have considerable activity against full length and T877A mutation carrying AR [37].

There is a recent research reporting newly identified surfaced exposed pocket on DNA binding domain of AR and targeting of this site considerably inhibited transcriptional activity of full-length AR and its splice variants [38]. Methylselenol and MDV3100 synergistically inhibited full length AR and AR-V7 in prostate cancer cells [39]. We will further discuss the role of enzalutamide and how one could potentially device strategies for overcoming enzalutamide resistance.

## Enzalutamide as an important AR-targeting agent

There are some exciting pieces of evidences obtained from in-vitro studies that have started to shed light on the underlying mechanisms of enzalutamide resistance. Significant inhibition of AR nuclear accumulation by enzalutamide was documented in a wide ranging cell culture and pre-clinical studies. However, in a recent study nuclear accumulation of full length AR was noted in androgen depleted PCa cells expressing either AR-V7 or ARv567es. Mice inoculated with AR-V7-knockdown cells were sensitive to enzalutamide induced tumor growth inhibition. Serial passage of the relapsed tumors in enzalutamide treated castrated mice gave rise to the tumors that were enzalutamide resistant. RNA-seq analysis revealed upregulated expression of ARv567es and AR-V7 in resistant tumors [40].

There is recent evidence suggesting that enzalutamide exposure induced glucocorticoid receptor (GR) expression in prostate cancer cells. Mechanistically it was shown that enzalutamide exerted growth inhibitory effects but co-treatment with Dexamethasone (Dex) reversed this growth inhibition. More importantly gene silencing of GR restored sensitivity of cancer cells to enzalutamide. Data obtained from ChIP-seq experiments indicated that AR bound DNA binding sites identified after DHT treatment were bound by GR after Dex treatment [41].

AZD5363 and enzalutamide have been shown to delay development of resistance against enzalutamide in preclinical models. Combinatorial approach effectively induced regression of tumor in xenografted mice at time of castration [42].

Enzalutamide exposure induced autophagic response in prostate cancer cells that operated through activation of AMP-dependent protein kinase (AMPK) and the suppression of mammalian target of rapamycin (mTOR). Gene silencing of AMPK inhibited autophagic response and induced apoptosis. Combinatorial approach consisting of enzalutamide and autophagy inhibitors notably inhibited tumor growth in mice orthotopically implanted with ENZA-resistant cells [43]. However, it has also been shown that enzalutamide was not effective in patients previously treated with docetaxel [44].

### Enzalutamide induced infiltration and metastasis

There is a direct piece of evidence emphasizing on the fact that Enzalutamide or bicalutamide increased macrophage infiltration in prostate cancer cells that resulted in an enhanced prostate cancer cell invasion. Mechanistically it was shown that Enzalutamide or bicalutamide downregulated AR-induced PIAS3 expression and enhanced pSTAT3-CCL2 signaling cascade. ASC-J9, an AR degradation enhancer exerted inhibitory effects on STAT3 phosphorylation/activation. CCR2 antagonist treatment also impaired Enzalutamide or bicalutamide mediated increase in cell invasion [45]. It has been experimentally verified that prostate cancer cells enhanced chemokine-CXCL9 induced recruitment of CD4(+) T cells than the surrounding normal prostate cells. Suppression of AR signals either through gene silencing or enzalutamide enhanced the recruitment of T cells [46].

### Improving the Efficacy of Enzalutamide by targeting of Twist and SRD5A

Twist is involved in epithelial-to-mesenchymal transition and metastasis and a therapeutic target. Co-treatment with Enzalutamide and vaccine directed against Twist resulted in considerably enhanced overall survival of TRAMP mice [47]. Steroid 5-alpha-reductases (SRD5A1 and SRD5A3) are overexpressed in different prostate cancer cell lines. Proliferation potential of SRD5A1 and SRD5A3 overexpressing cancer cells was notably reduced upon treatment with Dutasteride and Enzalutamide [48]. C2-substitution with benzyl and phenyl moieties is a remarkable advancement in improving efficacy of androgen receptor Pan-antagonists as evidenced by efficient tumor growth inhibition in xenografted mice [49].

Niclosamide, an FDA-approved antihelminthic drug efficiently induced AR-V7 protein degradation and AR-V7 mediated transcriptional activity [50,51]. Inhibition of allosteric site of the AR binding function 3 (BF3) using 1H-indole-2-carboxamides also exerted inhibitory effects on proliferation potential of enzalutamide-resistant prostate cancer cell lines [52]. In the following section, we will discuss further on the potential role of AR splice variants and therapeutic resistance.

## Androgen receptor variants in defining the complexity of PCa

It is becoming progressively more understandable that AR gene rearrangements stimulated expression of truncated AR variant proteins lacking the AR ligand-binding domain and synthesis of full-length AR was repressed. AR variant proteins tactfully induced AR transcriptional network in the absence of full-length AR or androgens [53]. It has been verified that castration induced marked increase in intratumoral androgen biosynthesis. Moreover, full-length AR and AR splice variants were also upregulated [54]. There is rapidly increasing scientific evidence emphasizing on the fact that androgen depletion induced AR splice variants (ARVs) in prostate cancer cells to retain basal AR activity. It was noted that androgen-bound AR exerted inhibitory effects on Androgen Receptor Variant-7 (AR-V7) expression by stimulating expression of proteins that suppressed AR-V7 expression. AR-V7 protein expression was low relative to AR-FL in androgen-deprived VCaP cells and mice xenografted with castration-resistant VCaP cancer cells [55]. However, previous intriguing piece of evidence emphasized on the fact that that some ARVs are functionally active independently in the absence of AR-FL to activate AR reporter constructs. Transfecting AR-negative DU145 cells independently with three ARVs truncated after exon 3 (AR-V1, mAR-V2 and AR-V7) revealed that AR-V7 and mAR-V4 (truncated after exon 4) indicated notable activity. Another important finding of the study was that gain of function of ARV7 was impaired in presence of dominant negative AR-V1 in PCa cells [56].

It has also been highlighted in a recent report that androgen depletion considerably enhanced interactions between splicing factors and AR pre-mRNA as evidenced by identification of two RNA splicing enhancers and their binding proteins ASF/SF2 and U2AF65 ([50,51]. AR8 lacks a DNA binding domain and is structurally different from other known AR splice variants. Surprisingly, AR8 localized primarily on the plasma membrane, probably through palmitoylated cysteine residues present within its C-terminal region. However, mutation of residues resulted in abrogation of post-translational modification and consequently loss of AR8 from plasma membrane. Considerably enhanced tyrosine phosphorylation of AR was noted in AR8 overexpressing prostate cancer cells upon treatment with EGF. Structurally, AR was noted to co-exist with EGFR and Src in EGF treated AR8 overexpressing prostate cancer cells [57].

Previously, it has been shown that microtubule-associated motor protein, dynein triggered nuclear import of AR. Moreover, overexpressing dynactin associated protein dynamitin in cancer cells considerably reduced dynein-cargo interaction and nuclear accumulation. Microtubule-interacting ARv567 was sensitive to taxane and docetaxel treatment was significantly effective in ARv567-expressing LuCap86.2 tumor xenografts [58]. AR and AR-V7 differentially activated target genes in co-operation with FOXA1 [59]. Circumstantial evidence also indicated that AR-Vs interacted with DNA independently of full-length AR in the absence of androgen to mediate a unique gene network [60]. Prostate cancer cells expressing constitutively active, C-terminally truncated low molecular weight AR-species lacking the AR-ligand binding domain (LBD) has added another layer of complexity in the targeting. Detailed mechanistic insights indicated that full length AR undergo heterodimerization with an ARΔLBD in androgen depleted prostate cancer cells. Stilbene and fluorinated dialkylaminostilbene (FIDAS) considerably inhibited AR activity in prostate cancer cells co-transfected with constitutively active ARΔLBD-variant Q640X and an AR-dependent reporter gene [61].

ARv567es, in which exons 5, 6, and 7 are deleted, is an AR variant notably involved in cancer progression. For a better understanding of the ARv567es mediated prostate cancer progression, probasin (Pb) promoter-driven ARv567es transgenic mouse was developed. Detailed analysis revealed that ARv567es induced epithelial hyperplasia after 16 weeks and invasive adenocarcinoma was noted after 1 year in mice [33,34]. AR-V7 mediated cancer progression has recently been studied in transgenic mouse model (AR3Tg) and results revealed that AR-V7 induced an expansion of prostatic progenitor cell population that consequently resulted in development of prostatic intraepithelial neoplasia. Additionally gene network associated with Epithelial to Mesenchymal transition was also noted to be activated [62].

It is also interesting to note that although gene network reported to be regulated by full length AR and C-terminally truncated variant of AR is overlapping, there are some genes which are exclusively triggered by AR variant, specifically, RHOB which is shown to be involved in the increased migration [63], and thus targeting RHOB could become a novel therapeutic strategy for PCa patients.

## The broader role of NF-κB signaling in PCa relevant to AR signaling

Classical NF-κB intracellular signaling operating through p65/p50 heterodimer is constitutively activate in prostate cancer cells. Noncanonical NF-κB pathway involves the processing of p100 to NF-κB2/p52 and prostate cancer cells chronically treated with Enzalutamide have been shown to display higher levels of NF-κB2/p52. NF-κB2 expressing LNCaP and C4-2B cells had a higher expression of AR-V7 splice variant. NF-κB2 silencing in VCaP and CWR22Rv1 cells significantly reduced AR-V7 splice variant expression [64]. It is interesting to note that nuclear factor-kappa B (NF-κB) signaling triggered expression of androgen receptor variants. Using a combinatorial anti-androgen and NF-κB-targeted therapy efficiently reduced tumor growth in xenografted mice [12,13].

## Activators and inhibitors of AR variants

Vav3, a Rho GTPase guanine nucleotide exchange factor is involved in enhancing transcriptional activity of AR3 and ARv567es. Interfering with Vav3 resulted in significantly reduced nuclear accumulation of AR3 and data obtained from co-immunoprecipitation assay revealed that Vav3 interacted with AR3 [65]. In accordance with this concept, another protein reported to inhibit activity of AR variant is FOXO1. In-depth analysis indicated that AKT mediated phosphorylation of FOXO1 exported it from nucleus thus allowing AR-V7 to transcriptionally upregulate expression of target genes. However, chemical inhibition of PI3K and AKT resulted in nuclear localization of FOXO1 that consequently inhibited AR-V7 activity [66]. Recently it has been reported that N-terminal tau5/AF5 domain (aa392-558) of AR is required for binding to Gli2, a transcription factor in the Hedgehog pathway. Confluence of information suggested that Gli2 C-Terminal Domain and Full Length Gli2 remarkably enhanced AR-V7 and AR567es transcriptional activity. Gli2 bound AR was noted at chromatin sites near to androgen responsive genes in Gli2 overexpressing LNCaP cells [9,10].

## Conclusion and perspectives

Emerging evidence as presented in this short review article clearly suggesting that the development of novel AR-targeting agents is not a distant possibility any more, and such developments are welcome news for PCa patients especially for those who develop CRPC and subsequent mCRPC after ADT. In the context of ADT especially the introduction of novel ADTs such as enzalutamide and abiraterone is a significant therapeutic advancement for the treatment of PCa; however, these agents are rapidly developing resistance largely contributed by the expression of AR variants and other resistance pathways. Therefore any developments of novel strategies for overcoming resistance to ADT or delaying and/or preventing the development of resistance would revolutionize in the better treatment of PCa patients after the surgical management.
